# *Clostridium sphenoides* Bloodstream Infection in Man

**DOI:** 10.3201/eid1701.101029

**Published:** 2011-01

**Authors:** Theodoros Kelesidis, Sotirios Tsiodras

**Affiliations:** Author affiliations: David Geffen School of Medicine at UCLA, Los Angeles, California, USA (T. Kelesidis);; University of Athens Medical School, Athens, Greece (S. Tsiodras)

**Keywords:** Clostridium sphenoides, bacteremia, bacteria, human infection, letter

**To the Editor:** The role of clostridia as intestinal pathogens has been recognized (*1*). However, the full extent of the pathogenicity, clinical spectrum, and optimal therapy of *Clostridium sphenoides* infections remains to be determined. We describe a case of bloodstream infection in a man that was caused by *C. sphenoides*.

A 68-year-old man was admitted to the hospital (Harbor UCLA Medical Center, Los Angeles, CA, USA) after a motor vehicle accident in December 2009. He was afebrile (temperature 37.2°C), was hemodynamically stable, and had generalized abdominal tenderness. Computed tomography scan of the abdomen and pelvis showed laceration of the spleen and focal aortic dissection at the aortic bifurcation. The patient underwent surgical exploration and splenic resection. No signs of bowel ischemia or laceration were identified during surgery. On the second day postoperation, he became hypotensive, and a fever of 39.2°C developed. Blood cultures (anaerobic bottles from 2 sets of blood cultures) grew a gram-positive rod, ultimately identified as *C. sphenoides*.

Bacterial growth was detected in the anaerobic bottles on day 4 of incubation by using the BacT/Alert system (bioMérieux, Marcy l’Etoile, France). The organism was subcultured to Brucella blood agar and was incubated anaerobically. On the basis of Gram staining and analysis of the organism’s morphologic features in culture (growth at 37°C, obligate anaerobe with spherical, subterminal spores and hemolytic colonies with irregular edges), its biochemical characteristics (the organism was motile, a citrate fermenter), and 16S rRNA gene sequencing, the organism was identified as *C. sphenoides*. The sequence obtained was 100% identical to a *C. sphenoides* 16S rRNA sequence (GenBank accession no. AB075772). Sensitivity testing with Etest showed that the isolate was susceptible to penicillin (MIC 0.094 μg/mL), ampicillin/sulbactam (MIC 0.125 μg/mL), metronidazole (MIC 1.5 μg/mL), and doripenem (MIC 0.5 μg/mL) and resistant to clindamycin (MIC 12 μg/mL).

The patient was given doripenem, 500 mg intravenously (IV) every 8 hours; metronidazole, 500 mg IV every 8 hours; and vancomycin, 1 gm IV every 12 hours. On the third day postoperation, blood cultures (anaerobic bottle from 1 set of blood cultures) again grew *C. sphenoides.* On the fourth day postoperation, he had persistent fever (38.5°C), became severely hypoxic, and was intubated. Repeat blood cultures were negative for *C. sphenoides*. A computed tomographic scan of the chest showed bilateral pneumonia, and a sputum culture grew *Serratia marcescens.* The patient underwent a 2-week course of doripenem and an 11-day course of metronidazole. He also received vancomycin for 7 days. The patient was eventually discharged to a rehabilitation facility after 2 weeks in the hospital.

*C. sphenoides* was initially thought not to be pathogenic in humans, but it has been occasionally reported as a human pathogen (Table) (*2**–**4*). The organism is sometimes acquired from food (*2*). Osteomyelitis (*3*) and peritonitis (*4*) caused by *C. sphenoides* have also been reported. The organism has characteristic biochemical properties, and citrate is a specific substrate for the isolation of *C. sphenoides* (*5*). The pathogenesis of *C. sphenoides* infections in humans remains unclear. *C. sphenoides* may produce small alterations on Vero cells in vitro, such as turning the cells oval without altering their size, and these changes are different from those caused by *C. difficile* (*6*).

An unusual aspect of the infection in our patient was that it represents a primary invasion of clostridia in apparently healthy colonic tissue. Ordinarily, the absolute prerequisite for clostridial infection is a focus of necrotic tissue, which this organism then infects (*7*). Reports of invasion without an evident necrotic focus (or a probable focus as in a cancer) are rare (*4*). More studies are needed to clarify the pathogenesis of *C. sphenoides* infections in humans.

Persistent bacteremia over >48 hours (second and third days postoperation) indicates that *C. sphenoides* was a true pathogen and the cause of bloodstream infection, rather than an apathogenic member of the colonic flora. MacLennan isolated *C. sphenoides* from war wounds (*7*). In the original report of the discovery of *C. sphenoides* (*8*), medical aspects were referred to unpublished data, and it is not possible to tell whether the 3 patients from whom the organism was isolated in pure culture had gas gangrene or whether simple wound surface colonization was being reported.

We could not identify any report of solitary *C. sphenoides* infection. The organism is extremely uncommon in human feces (*9*) and has been found in only 4% of soil samples (*10*).

In 1 study, *C. sphenoides* was isolated from 2 (6%) of 19 stool samples from children without diarrhea (*6*). These 2 isolates were sensitive to most antimicrobial drugs, including amoxicillin, ampicillin, aztreonam, ceftriaxone, chloramphenicol, and penicillin G (*6*). However, data on susceptibilities of *C. sphenoides* to various antimicrobial agents are lacking. We report susceptibility of *C. sphenoides* to carbapenems and metronidazole and resistance to clindamycin.

The full extent of the pathogenicity, clinical spectrum, and optimal therapy of *C. sphenoides* infections remain to be determined. Clinicians should be aware of the possible pathogenic role of *C. sphenoides* in humans.

**Table:** Descriptions of cases of infection with Clostridium sphenoides*

Authors, year, and reference	Country	Patient age, y/ sex	Underlying conditions/ risk factors	Signs and symptoms	Microbiologic findings	Treatment	Outcome
Sullivan et al., 1980 (*2*)	Canada	39/F	None. Ate Chinese food 8 h before onset of symptoms	Severe abdominal cramps and diarrhea	*C. sphenoides* isolated from stool culture. Susceptibility testing to antimicrobial drugs not reported	No antimicrobial drugs given	Spontaneous recovery within 96 h of onset of illness
Isenberg et al., 1975 (3)	USA	13/M	None. Trauma at the area of osteomyelitis 1 y before diagnosis	Osteomyelitis	*C. sphenoides* isolated from bone culture. Sensitive to penicillins, cephalosporins, chloramphenicol, tetracyclines, macrolides; resistant to aminoglycosides, polymyxins	Phenethicillin 2 g IV daily for 3 d, followed by 1 g IV daily for 3 mo	Recovered. No evidence of disease clinically or radiologically after 3 y of follow up
Felitti, 1970 (*4*)	USA	6/F	Chronic neutropenia, lifelong history of recurrent attacks of otitis media, oral ulcers, periodontal abscesses, chronic gingivitis	Fever, abdominal cramps, occasional vomiting, peritonitis	*C. sphenoides* isolated from the peritoneum. Blood cultures negative. Susceptibility testing to antimicrobial drugs not reported	No antimicrobial drugs given	Died

*IV, intravenously.

## References

[1] Clostridia as intestinal pathogens. Lancet. 1977;2:1113–4. 73015

[2] Sullivan SN, Darwish RJ, Schieven BC. Severe diarrhea due to *Clostridium sphenoides*: a case report. Can Med Assoc J. 1980;123:398. 7260781 PMC1704784

[3] Isenberg HD, Lavine LS, Painter BG, Rubins WH, Berkman JI. Primary osteomyelitis due to an anaerobic microorganism. Am J Clin Pathol. 1975;64:385–8. 1163490 10.1093/ajcp/64.3.385

[4] Felitti VJ. Primary invasion by *Clostridium sphenoides* in a patient with periodic neutropenia. Calif Med. 1970;113:76–8. 5457520 PMC1501556

[5] Walther R, Hippe H, Gottschalk G. Citrate, a specific substrate for the isolation of *Clostridium sphenoides.* Appl Environ Microbiol. 1977;33:955–62. 869540 10.1128/aem.33.4.955-962.1977 PMC170796

[6] Ferreira CE, Nakano V, Avila-Campos MJ. Cytotoxicity and antimicrobial susceptibility of *Clostridium difficile* isolated from hospitalized children with acute diarrhea. Anaerobe. 2004;10:171–7. 10.1016/j.anaerobe.2004.02.003 16701515

[7] MacLennan JD. Anaerobic infections of war wounds in the Middle East. Lancet. 1943;2:94. 10.1016/S0140-6736(00)87071-3

[8] Report of the Committee upon Anaerobic Bacteria and Infections. Medical Research Council Special Report Series, Great Britain,#39, 1919. http://www.archive.org/stream/cu31924000323687/cu31924000323687_djvu.txt

[9] Kahn MC. Anaerobic spore bearing bacteria of the human intestine in health and in certain diseases. J Infect Dis. 1924;35:423. 10.1093/infdis/35.5.423

[10] Lindberg RB. The bacterial flora of battle wounds at the time of the primary debridement. Ann Surg. 1955;141:369. 10.1097/00000658-195503000-00012 14350578 PMC1609687

